# ING4 suppresses tumor angiogenesis and functions as a prognostic marker in human colorectal cancer

**DOI:** 10.18632/oncotarget.12984

**Published:** 2016-10-27

**Authors:** Yansu Chen, Yefei Huang, Pingfu Hou, Zhe Zhang, Yafei Zhang, Weimin Wang, Guixiang Sun, Lichun Xu, Jianwei Zhou, Jin Bai, Junnian Zheng

**Affiliations:** ^1^ Jiangsu Key Laboratory of Biological Cancer Therapy, Xuzhou Medical University, Xuzhou 221002, Jiangsu Province, China; ^2^ School of Public Health, Xuzhou Medical University, Xuzhou 221002, Jiangsu Province, China; ^3^ Jiangsu Center for the Collaboration and Innovation of Cancer Biotherapy, Cancer Institute, Xuzhou Medical University, Xuzhou 221002, Jiangsu Province, China; ^4^ Department of Oncology, Yixing People's Hospital, Yixing 214200, Jiangsu Province, China; ^5^ Department of Molecular Cell Biology and Toxicology, Cancer Center, School of Public Health, Nanjing Medical University, Nanjing, 211166, China

**Keywords:** ING4, Sp1, angiogenesis, colorectal cancer, prognosis

## Abstract

ING4, a potential tumor suppressor, is implicated in cell cycle arrest, apoptosis, cell migration and angiogenesis. Here, we investigated the clinical value of ING4 and its impact on angiogenesis in colorectal cancer (CRC). In this study, we found that ING4 expression was significantly reduced in CRC tissues versus paired normal colon tissues. Moreover, low ING4 expression was significantly associated with increased lymph node metastasis, advanced TNM stage and poor overall survival. Multivariate Cox regression analysis showed that ING4 expression was an independent favourable prognostic factor for CRC (hazard ratio = 0.45, *P* = 0.001). In addition, we found that ING4 strongly inhibited CRC angiogenesis by suppressing Sp1 expression and transcriptional activity through ubiquitin degradation and down-regulating the expressions of Sp1 downstream pro-angiogenic genes, MMP-2 and COX-2. Moreover, ING4 might inhibit phosphorylation activity of cyclin/CDK2 complexes to trigger Sp1 degradation by inducing p21 expression in despite of p53 status. Our findings imply that reduced ING4 expression in CRC resulted in increased angiogenesis and contributed to CRC metastasis and poor prognosis. Restoration of ING4 may be a novel strategy for the treatment of metastatic CRC.

## INTRODUCTION

Colorectal cancer (CRC) is the most common malignancy with the third largest incidence and mortality among all diagnosed cancers in the worldwide[1]. The death rate has a significant reduction due to increased use of sigmoidoscopy and colonoscopy with polypectomy in the USA and several other high-income countries, but a rapidly increase trend in China [1, 2]. The metastatic diseases are the main cause for the high mortality rates in CRC patients. The 5-year relative survival of CRC patients is 90.1% for these with localized stage, and drops to 69.2% and 11.7%, respectively, once patients have regional spread or distant metastases [3].

Solid tumor metastasis is a sequential multi-steps, and angiogenesis is widely believed to be a critical step for metastasis [4]. The molecular mechanisms underlying CRC angiogenesis have been validated to be clinically important because of their relation to the prognosis and treatment response of the patients [5, 6]. Therefore, great efforts to unravel the mechanisms driving this process are required for providing novel biomarkers for prognosis and future therapeutic interventions.

Specificity protein 1 (Sp1), a well-known member of the transcript factors' family, can directly bind to the promoters of some responsive target genes through the GC-rich putative DNA-binding domain to promote transcription [7]. Evidences exist that both Sp1 expression and transcriptional activity are excessively increased in various types of cancers, and high expression of Sp1 is generally considered as a negative prognostic factor [8]. Moreover, activation of Sp1 is implicated in an ample variety of cancer biological processes, including sustained proliferation, replicative immortality and induction of angiogenesis, invasion and metastasis [8, 9]. However, Sp1 activity is highly regulated by some post-translational modifications, including phosphorylation, O-linked glycosylation, acetylation, SUMOylation, and ubiquitylation, and at last is targeted to proteasome-mediated degradation pathways [7]. Therefore, exploring how Sp1 is aberrant activated is of great importance for the understanding of tumor progression.

Cell cycle protein, inhibitor of growth protein 4 (ING4), one member of INGs, possess a common schematic structure, including a Plant Homeo Domain, a Nuclear Localisation Signal and a Novel Conserved Region [10]. ING4 has been identified as an important tumor suppressor gene, which is involved in cell cycle arrest, apoptosis, DNA repair, chromatin modification, inhibiting cell migration and angiogenesis [10–12]. Recent studies have indicated that both mRNA and protein levels of ING4 are lost or decreased in CRC when compared with normal colon tissues [13, 14]. Moreover, decreased ING4 expression in CRC is associated with increased microvessel density [14], however, the clinical value of ING4 for CRC patients and precise mechanism of ING4 in CRC angiogenesis has not yet been described. In this study, using a retrospective CRC patients' cohort and a series of *in vitro* and *in vivo* experiments, we aimed to explore the biological function and clinical significance of ING4 in CRC.

## RESULTS

### ING4 expression is reduced in CRC versus normal colon tissues

**Figure 1:**
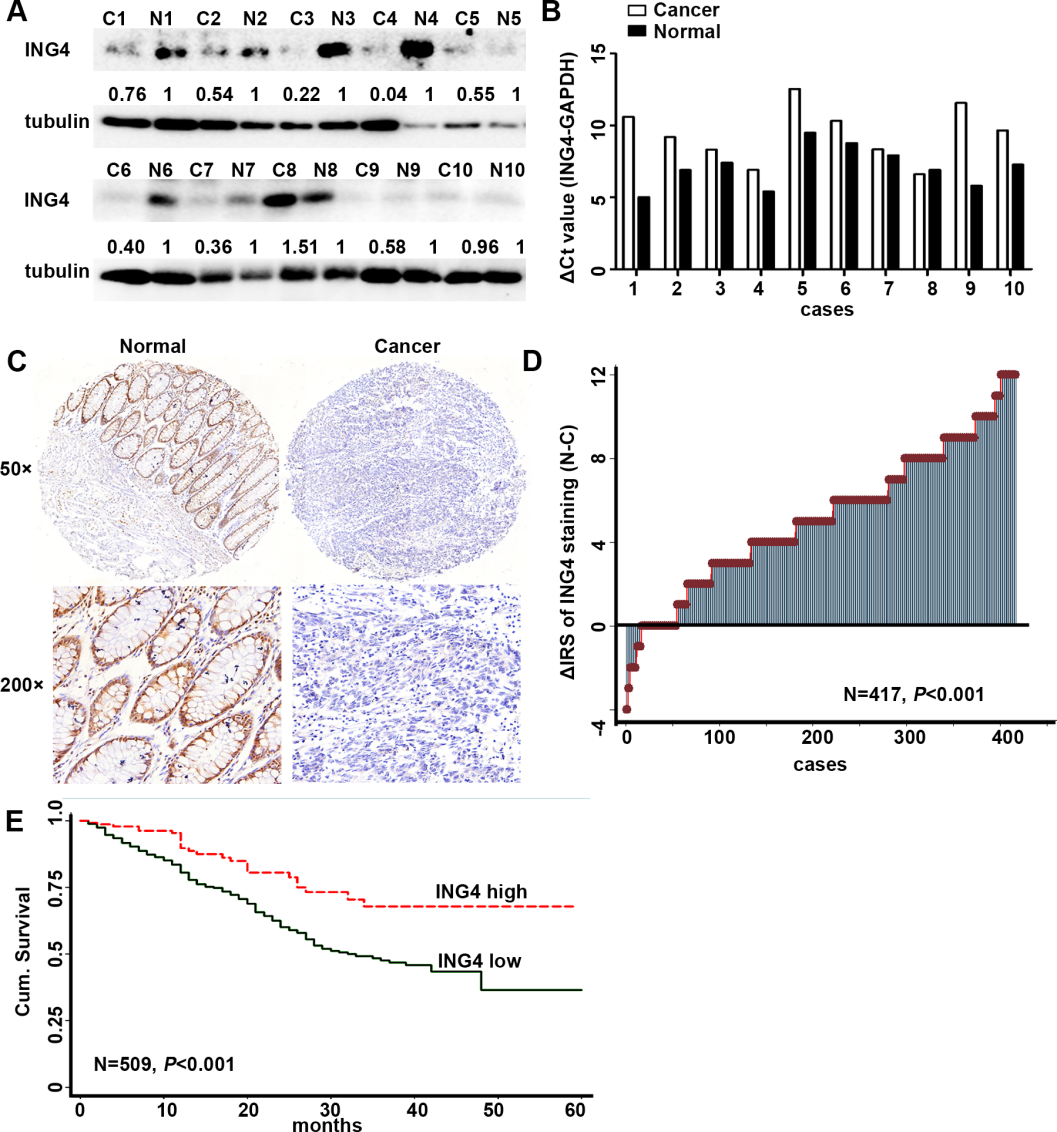
Expression of ING4 was reduced in human CRC and positively associated with overall survival in CRC patients (**A**) Determination of ING4 protein levels in 10 cancer tissues and paired non-cancerous normal colon tissues by western blot. (**B**) Real time PCR showed the differences of the raw delta Ct values of ING4 amplification after normalization by GAPDH in cancers and paired normal tissues. (**C**) Representative images of ING4 immunohistochemical staining in TMAs were shown. Note: Top panel: original magnification, × 50; bottom panel: original magnification, **×** 200. (**D**) The distribution of the difference in staining intensities of ING4 in CRC tissues compared with that in paired normal tissues. C, CRC tissues; N, paired non-cancerous colon tissues. (**E**) Kaplan–Meier curves showed overall survival of CRC according to expression levels of ING4.

To detect the expression level of ING4, 10 pairs of human fresh CRC tissues and the paired normal non-cancerous colon tissues were used. The results of western blot showed that ING4 expression was dramatically reduced in 9 of 10 (90%) cancers when compared with the normal tissues (Figure 1A). Simultaneously, real time PCR was performed to test the mRNA expression levels of ING4, and the raw threshold cycle (delta Ct) values of ING4 amplification after normalization by GAPDH were shown in Figure 1B. Accordant with the results of western blot, 9 of 10 (90%) cancer tissues had higher delta Ct values of ING4 amplification compared with normal tissues, which meant that ING4 expression was down-regulated in cancers. Moreover, the CRC TMAs including the cancer and corresponding normal tissues were used to further validate these findings. The results of IHC indicated that ING4 was mainly located in the nucleus (Figure 1C), and ING4 IRS was significantly lower in 363 out of 417 (87%) cancers than the normal tissues (Figure 1D, *P* < 0.001).

### Low ING4 expression correlates with clinicopathological parameters and poor overall survival in CRC patients

Fisher's exact test was used to examine the correlation of ING4 expression in cancer with clinicopathological characteristics. The data revealed that ING4 expression was significantly negatively associated with the lymph node metastasis and TNM stages (Table 1, *P* < 0.001). Moreover, our results showed that there were marginal correlations of ING4 expression with the depth of invasion (*P* = 0.072) and distant metastasis (*P* = 0.074). But, ING4 expression was not associated with age, sex, tumor diameter and the differentiation.

**Table 1:** Relationship between the expression level of ING4 and clinicopathological features of CRC patients

Variables	ING4 expression (*n*=509 cases)		
	Low (%)	High (%)	*P*^a^
All patients	359 (100)	150 (100)	
Age(years)			1.000
≤ 65	147 (41)	62 (41)	
>65	212 (59)	88 (59)	
Gender			0.280
Males	211 (59)	80 (53)	
Females	148 (41)	70 (47)	
Depth of invasion*			0.072
T1/T2	14 (4)	12 (8)	
T3/T4	340 (96)	132 (92)	
Lymph node metastasis			<0.001
N0	207(58)	115 (77)	
N1/N2/N3	152 (42)	35 (23)	
Distant metastasis^#^			0.074
M0	340(95)	148 (99)	
M1	17 (5)	2 (1)	
TNM stage			<0.001
I	41 (11)	28 (19)	
II	150 (42)	92 (61)	
III	150 (42)	27 (18)	
IV	18 (5)	3 (2)	
Tumor diameter			0.592
≤5 cm	258 (72)	104 (69)	
>5cm	101 (28)	46 (31)	
Differentiation^$^			0.692
Poor	57 (16)	26 (18)	
Moderate/high	299 (84)	120 (82)	

a Two-sided Fisher's exact tests.

* The depth of invasion of cancer in 11 patients cannot be assessed.

# The distant metastasis of cancer in 2 patients cannot be assessed.

$ We lost the data of 7 CRC patients.

The Kaplan-Meier survival curve and the log-rank test were applied to explore whether ING4 expression was correlated with overall survival. Our results demonstrated that CRC patients with low ING4 expression had a worse overall survival than the ones with high ING4 expression (Figure 1E). The survival rate dropped from 68% in patients with high ING4 expression to 37% in those with low ING4 expression. To further evaluate the prognostic value of ING4 expression, the univariate and multivariate Cox regression analyses were performed. The univariate Cox regression analysis showed that gender, lymph node metastasis, depth of invasion, distant metastasis, TNM stage and ING4 expression, except for age, histological type and tumor diameter, were the prognostic factors for the CRC patients (Table 2). The multivariate Cox regression analysis went on declaring that ING4 expression was an independent prognostic biomarker for the CRC patients after adjusting with age, gender, histological type, tumor diameter and TNM stage (HR = 0.45, 95% CI = 0.29–0.71, *P* = 0.001, Table 3).

**Table 2:** Univariate Cox regression analysis of ING4 expression and clinicopathological variables predicting the survival of CRC patients

Variables	(*n* = 509 cases)	
	HR (95% CI)	*P*
Age (≤ 65 vs. > 65)	1.35 (0.98–1.85)	0.066
Gender (Males vs. Females)	1.39 (1.03–1.89)	0.033
Lymph node metastasis (N0 vs. N1/N2/N3)	1.42 (1.05–1.94)	0.024
Depth of invasion(T1/T2 vs. T3/T4)	7.00 (1.73–28.3)	0.006
Distant metastasis (M0 vs. M1)	2.31 (1.18–4.53)	0.015
TNM stage (I–II vs. III/IV)	1.48 (1.09–2.01)	0.011
Histological type (Moderate/high vs. Poor)	1.36 (0.94–1.99)	0.101
Tumor diameter (≤ 5 cm vs. > 5 cm)	1.21 (0.87–1.70)	0.246
ING4 expression (Low vs. High)	0.46 (0.30–0.70)	< 0.001

Abbreviations: HR: hazard ratio; CI: confidence interval.

**Table 3:** Multivariate Cox regression analysis models assessing the effects of covariates on OS in CRC patients

Variables	(*n* = 509 cases)	
	HR (95%CI)	*P*
Age (≤ 65 vs. > 65)	1.29 (0.93–1.80)	0.120
Gender (Males vs. Females)	1.50 (1.10–2.06)	0.011
Histological type (Moderate/high vs. Poor)	1.35 (0.92–1.98)	0.123
Tumor diameter (≤ 5 cm vs. > 5 cm)	1.24 (0.88–1.74)	0.209
TNM stage (I–II vs. III/IV)	1.40 (1.01–1.92)	0.041
ING4 expression (Low vs. High)	0.45 (0.29–0.71)	0.001

Abbreviations: HR: hazard ratio; CI: confidence interval.

### ING4 suppresses CRC angiogenesis *in vitro* and *in vivo*

**Figure 2:**
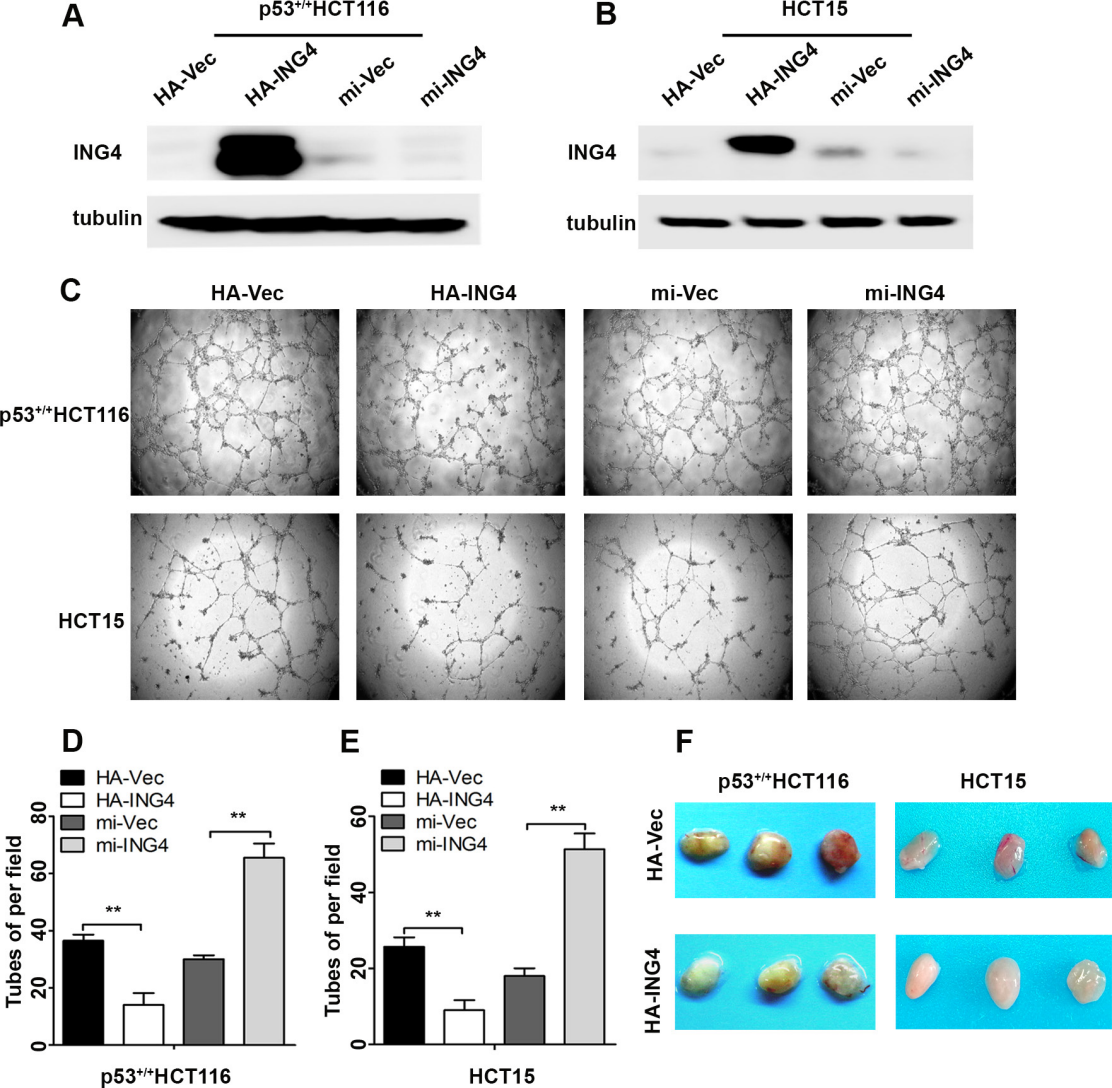
Expression of ING4 in CRC cells negatively regulated tube formation *in vitro* and inhibited blood vessel formation *in vivo* (**A**–**B**) Western blot was used to test the ING4 expression in the p53^+/+^HCT116 and HCT15 cells with ING4 overexpression (HA-ING4), ING4 knockdown (mi-ING4) and respective vector control (HA-Vec and mi-Vec). (**C**) ING4 in CRC cells negatively regulated tube formation. (**D**–**E**) Numbers of complete tubular structures formed by HUVECs were counted for ING4 overexpressed, knocked down and control groups (*n* = 3/group) in CRC cells. (**F**) Photographs of matrigel plugs with ING4 overexpressed or control p53^+/+^HCT116 and HCT15 cells excised from mice after 10 days of growth *in vivo*. Data are presented as means ± standard deviations. ^*^P < 0.05, ^**^P < 0.001 (Student's *t*-test).

Since our CRC cohort showed that ING4 expression was associated with metastasis, and angiogenesis is widely believed to be an important step for tumor metastasis [4], here we further study the function of ING4 expression in CRC angiogenesis. Two CRC cell lines, p53^+/+^HCT116 and HCT15 were overexpressed or knocked down ING4 (Figure 2A–2B), then the conditioned medium was collected to perform the tube formation assays *in vitro*. Our data showed that the average number of complete tubular structures formed by HUVECs had a significant decrease in the conditioned medium collected from ING4 overexpressed cells but a significant increase in that collected from ING4 knocked down cells, when compared with corresponding controls, respectively (Figure 2C–2E).

Furthermore, to investigate the suppressive role of ING4 in angiogenesis, the *in vivo* matrigel plug was performed. The ING4 overexpressed or control p53^+/+^HCT116 and HCT15 cells mixing with matrigel were injected subcutaneously into the nude mice, respectively, and then the neovessel formation was detected. The visual examination revealed matrigel plugs containing ING4 overexpressed cells had distinctly reduced neovessels when compared with those plugs with control cells (Figure 2F).

### ING4 regulates Sp1 expression and transcriptional activity to inhibit the expressions of its downstream target pro-angiogenic genes and CRC angiogenesis

**Figure 3:**
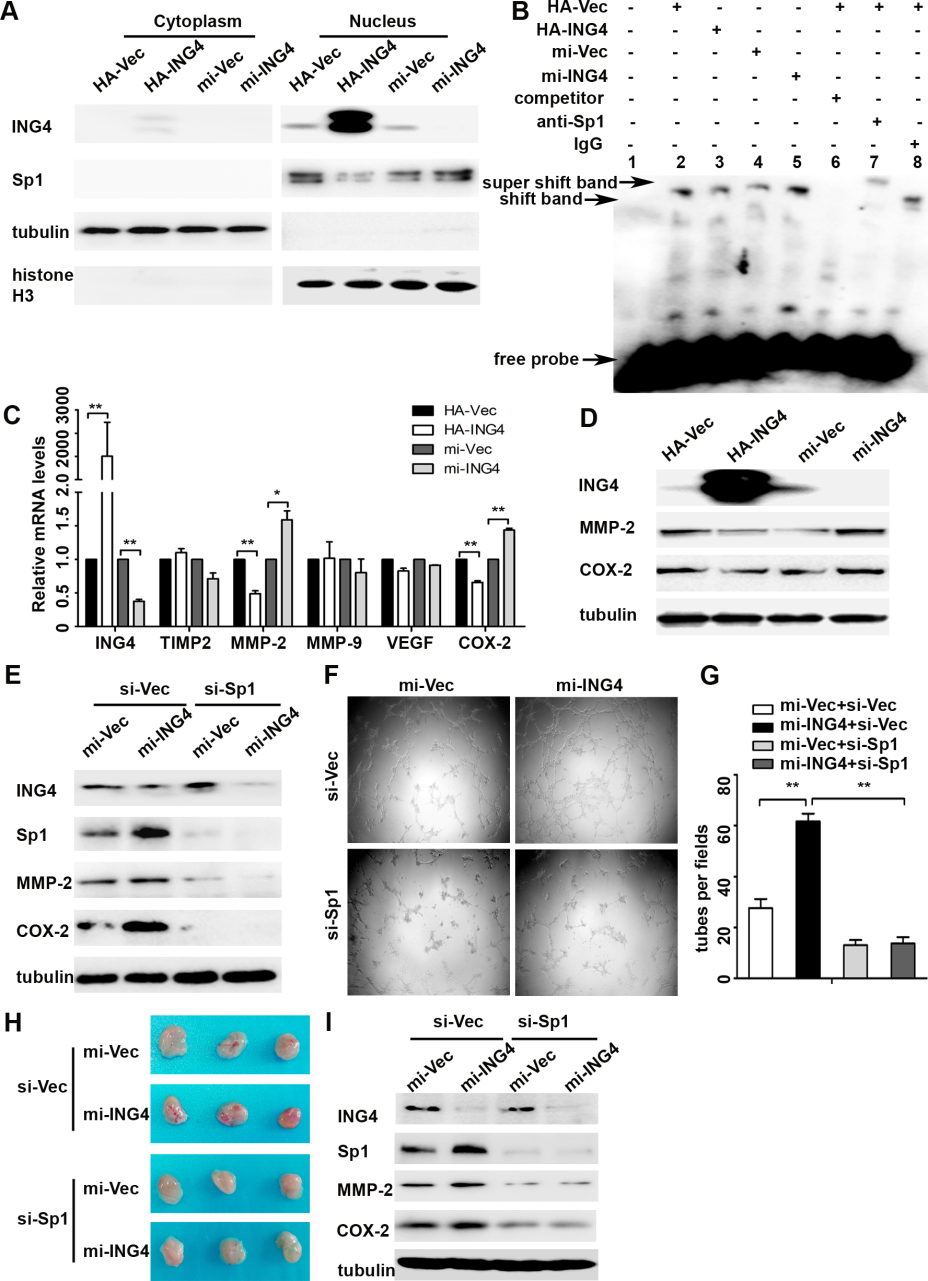
ING4 suppressed Sp1 expression and transcriptional activity to regulate expressions of its target pro-angiogenic genes MMP-2 and COX-2 and angiogenesis *in vitro* and *in vivo* (**A**) Western blot confirmed that ING4 inhibited the expression of nuclear Sp1. (**B**) ING4 altered the DNA affinity of Sp1. EMSA was performed using nuclear protein extracts from p53^+/+^HCT116 transfected with ING4 overexpression (HA-ING4), knockdown (mi-ING4) and respective controls plasmids (HA-Vec and mi-Vec). Lane 1 contains no nuclear extracts. All other lanes contain 1μg nuclear extracts. Lane 6 represents competition analysis using 100-fold unlabeled Sp1 probes. The super shift band was observed when the Sp1 antibody was added (lane 7) and IgG was used as negative control for super shift (lane 8). (**C**–**D**) Real time PCR and western blot were used to explore the expressions of Sp1 downstream pro-angiogenic genes MMP-2 and COX-2 in ING4 over-expressed, knocked down and control p53^+/+^HCT116 cells. (**E**) The increased expressions of MMP-2 and COX-2 by ING4 knockdown were abolished by Sp1 siRNA (si-Sp1). (**F**) Conditioned medium was collected and applied in tube formation. (**G**) Numbers of complete tubular structures formed by HUVECs were counted for ING4 knockdown, Sp1 knockdown or co-knockdown and control groups in p53^+/+^HCT116 (*n* = 3/group). (**H**) Photographs of matrigel plugs with ING4 knockdown, Sp1 knockdown or co-knockdown and control p53^+/+^HCT116 cells excised from mice after 10 days of growth *in vivo*. (**I**) The expressions of ING4, Sp1, MMP-2 and COX-2 were examined by western blot in matrigel plugs containing ING4 knockdown, Sp1 knockdown or co-knockdown and control p53^+/+^HCT116 cells. Data are presented as means ± standard deviations. ^*^P < 0.05, ^**^P < 0.001 (Student's *t*-test).

Studies have reported that activation of Sp1 plays a promotive role in tumor angiogenesis [8]. Here, we investigated whether ING4 could inhibit Sp1 transcriptional activity to exert its suppressive effect on tumor angiogenesis. Our data showed that the expression of nuclear Sp1 was dramatically reduced in the ING4 overexpressed p53^+/+^ HCT116 cells, whereas increased in the ING4 knockdown cells when compared with the corresponding controls (Figure 3A). Simultaneously, EMSA was performed to determine whether ING4 affected the Sp1-binding affinity to a defined Sp1-responsive DNA-binding element. It was showed that the DNA-protein binding was sequence specific and this binding was eliminated by 100-fold unlabeled Sp1 competitor probe. When Sp1 antibody was added, a specific super shift band was formed, while no super shift complex was observed when a control IgG was substituted. Furthermore, our data also indicated that ING4 overexpression led to an apparent decrease of Sp1-binding affinity, whereas ING4 knockdown increased this binding when compared with the respective controls (Figure 3B).

Next, our data also showed that ING4 overexpression significantly reduced the mRNA expressions of Sp1 downstream pro-angiogenic genes, MMP-2 and COX-2, by 52% and 35%, respectively, while ING4 knockdown significantly increased these gene mRNA expression by 1.6 and 1.4 fold, respectively (Figure 3C). According with the findings in the mRNA levels, the results of protein expression also showed that ING4 overexpression inhibited MMP-2 and COX-2 expression, but ING4 knockdown enhanced their protein expression versus the corresponding controls (Figure 3D).

To confirm MMP-2 and COX-2 were the responsive target genes of Sp1 in ING4 regulating CRC angiogenesis, we used siRNA to silence Sp1 expression in the ING4 knocked down p53^+/+^HCT116 cells, and then collected the conditioned medium to test the impact on angiogenesis *in vitro*. Our data revealed that increased MMP-2 and COX-2 expressions in the ING4 knocked down cells were distinctly reduced in both Sp1 and ING4 knocked down cells (Figure 3E). The data of tube formation assay *in vitro* also showed that the ING4-knockdown-induced tube formation was significantly abolished by inhibition of Sp1 (Figure 3F–3G). Moreover, in order to verify the findings *in vitro*, ING4 and Sp1 knocked down respectively or together and control p53^+/+^HCT116 cells were mixed with matrigel and injected subcutaneously into the nude mice, respectively, then the neovessel formation was detected. The visual examination revealed ING4-knockdown-elevated neovessels in matrigel plugs could be abrogated by Sp1 silence (Figure 3H). Simultaneously, western blot showed significant reduced expression levels of MMP-2 and COX-2 in the matrigel plugs of the ING4 and Sp1 knockdown together group compared with the ones of the ING4 knockdown alone group (Figure 3I).

### ING4 promotes ubiquitin-mediated Sp1 degradation to inhibit Sp1 expression and transcriptional activity

**Figure 4:**
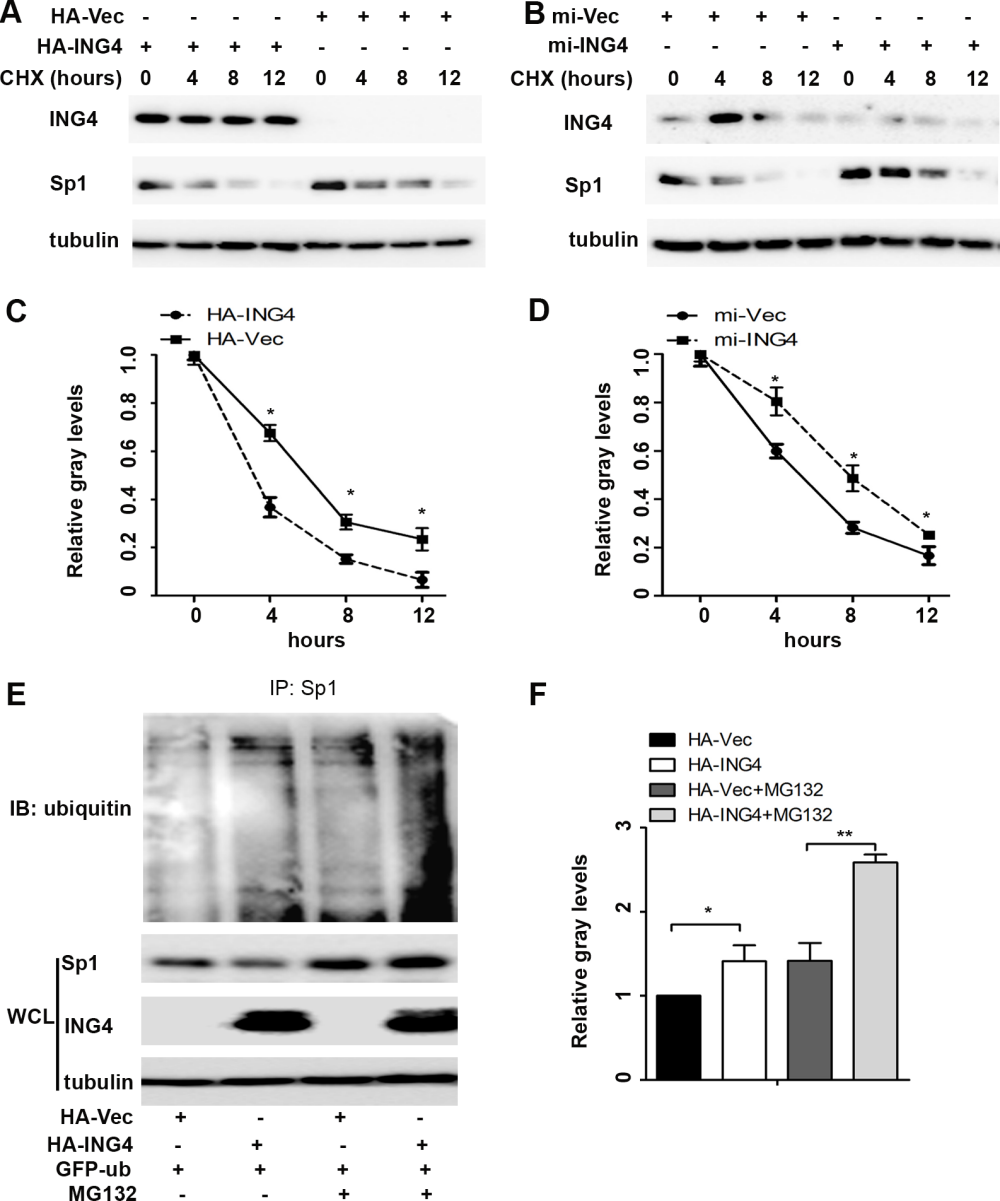
ING4 promoted Sp1 ubiquitin degradation (**A**–**B**) ING4 overexpression (HA-ING4) significantly enhanced but ING4 knockdown (mi-ING4) decreased the degradation of Sp1. p53^+/+^HCT116 cells were transfected with ING4 overexpression, knockdown and respective controls plasmids 48 hours, respectively, followed by exposure to cycloheximide (CHX; 50 mg/ml) for 0, 4, 8 or 12 hour, and the whole-cell lysates were detected by western blot. (**C**–**D**) The intensity of the Sp1 protein bands was analyzed by densitometry, after normalization to the corresponding beta-actin level. (**E**) Ubiquitination of Sp1 was induced by ING4. The p53^+/+^HCT116 cells were transfected with GFP-ub together with HA-Vec or HA-ING4 plasmids for 48 hour and then pretreated with MG132 (10 μM) for 6 hour, and these whole-cell lysates were immunoprecipitated using anti-Sp1 antibody, and ubiquitination was detected with ubiquitin antibody. Endogenous Sp1 were examined by western blot. (**G**) The intensity of the Sp1 ubiquitin bands was analyzed by densitometry. The data are means ± standard deviations from three independent experiments. ^*^P < 0.05, ^**^P < 0.001 (Student's *t*-test).

Various studies have suggested that Sp1 expression and transcriptional activity are largely dependent on its gene expression and the protein stability [8]. So, we firstly examined the Sp1 mRNA levels and found no change in the mRNA levels regardless of ING4 expression (Supplementary Figure S1). Next, we further investigated whether ING4 affected Sp1 protein stability. To determine this, we treated p53^+/+^HCT116 cells with cycloheximide, an inhibitor of protein synthesis, and found that the speed of Sp1 degradation was significantly accelerated by ING4 overexpression whereas slowed down by ING4 knockdown (Figure 4A–4D) when compared with the corresponding controls. These data suggested that ING4 could regulate Sp1 instability to control its expression and transcriptional activity.

Accumulated evidences have displayed that at last the ubiquitin-dependent proteasome will program to degrade instable Sp1 protein [7]. To elucidate this, p53^+/+^HCT116 cells were treated with the proteasome inhibitor MG132, it was showed that the Sp1 ubiquitylation was significantly increased in the ING4 overexpression cells, and the loss of Sp1 in ING4 overexpression cells was distinctly inhibited by MG132, when compared with the corresponding control (Figure 4E–4F).

### ING4 requires p21 to mediate instability of Sp1 regardless of p53 status

**Figure 5:**
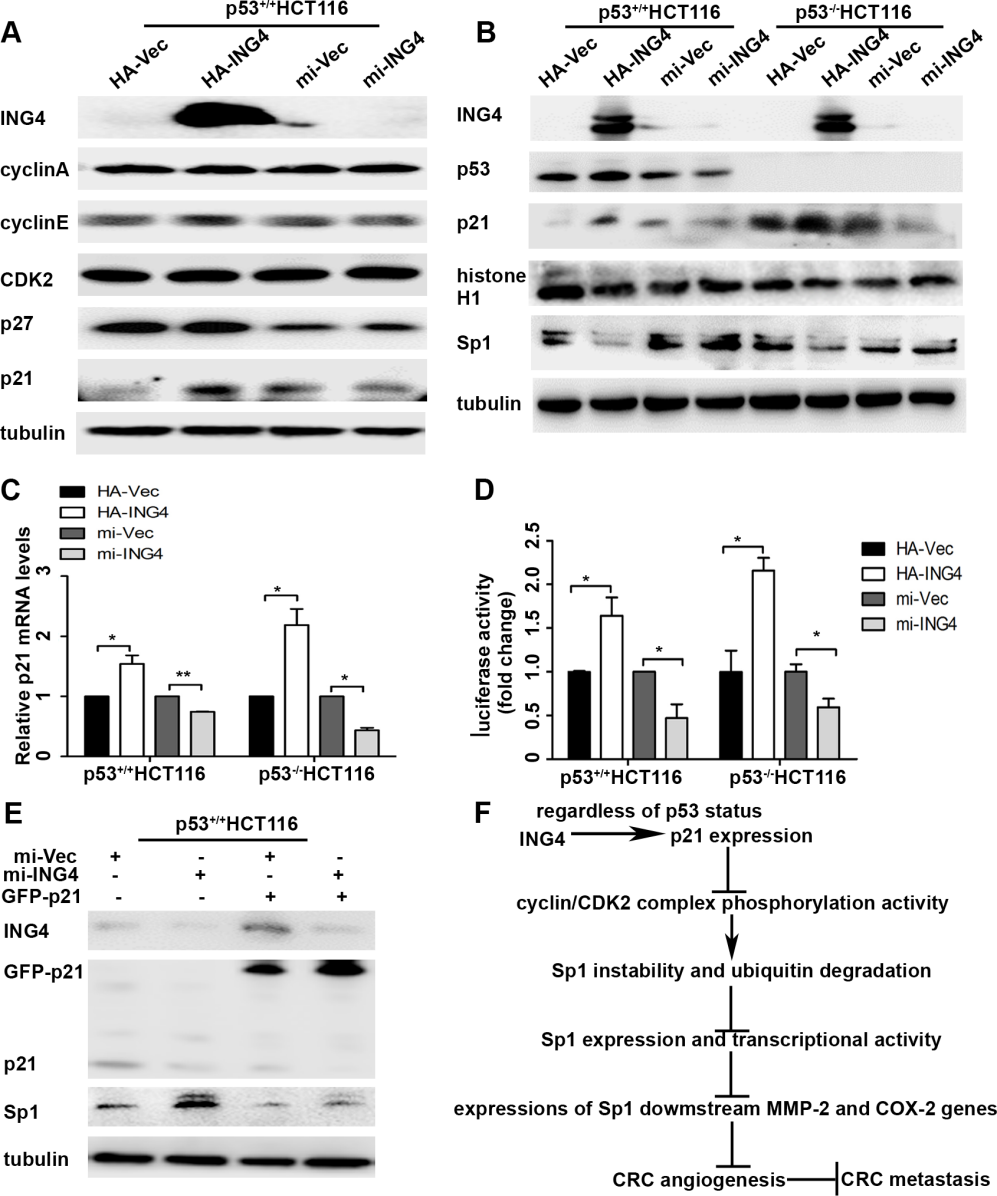
ING4 regulated p21 expression to mediate the instability of Sp1 (**A**) ING4 increased p21 expression. The expressions of cell-cycle associated proteins, such as cyclin A, cyclin E, CDK2, p21 and p27 in the p53^+/+^HCT116 cells with ING4 overexpression (HA-ING4) or knockdown (mi-ING4) and the respective controls (HA-Vec and mi-Vec). (**B**) ING4 regulated expressions of p21 and Sp1 regardless of p53 status. The expressions of p53, p21, Sp1 and histone H1, a sensitive protein for cyclin/CDK2 complexes phosphorylation activity, in the p53^+/+^HCT116 and p53^−/−^HCT116 cells with ING4 overexpression or knockdown and the respective controls. (**C**) ING4 regulated p21 mRNA expression. The mRNA expressions of p21 in the p53^+/+^HCT116 and p53^−/−^HCT116 cells with ING4 overexpression or knockdown and the respective controls was determined by real time PCR. (**D**) ING4 regulated p21 promoter activity. The reporter gene assay was used to test the p21 promoter activity in the p53^+/+^HCT116 and p53^−/−^HCT116 cells with ING4 overexpression or knockdown and the respective controls. (**E**) ING4 regulated p21 to mediate Sp1 expression. The expressions of p21 and Sp1 were tested in the p53^+/+^HCT116 cells transfected with p21 overexpression (GFP-p21) together with ING4 knockdown and the control plasmids. (**F**) The model of ING4 suppressing tumor angiogenesis through p21/cyclin/CDK2 complexes/Sp1/MMP-2 and COX-2 signaling pathway. Data are presented as means ± standard deviations.^*^P < 0.05, ^**^P < 0.001 (Student's *t*-test).

It is known that Sp1 phosphorylation modification plays a leading role in changes of its stability, and cell cycle signaling kinases have been shown to involve in this process [15]. Here we investigated whether ING4 could regulate cell cycle proteins, cyclin/cyclin-dependent kinase 2 (cyclin/CDK2) complexes and their inhibitors p21 and p27 to affect Sp1 stability. Our data revealed that p21 expression was up-regulated by ING4 overexpression, whereas down-regulated by ING4 knockdown. There was no change in the expressions of cyclin A, cyclin E, CDK2 and p27 (Figure 5A).

Studies have made clear that p21 expression can be regulated through p53 dependent or independent signaling pathway [10, 16]. To examine whether ING4 regulated p21 expression was dependent on p53 expression, the p53^+/+^HCT116 and p53^−/−^HCT116 cells were used. Our data showed that regardless of p53 status, ING4 could positively regulate p21 expression, and the expressions of Sp1 and histone H1, a sensitive protein for cyclin/CDK2 complexes phosphorylation activity [17], were dramatically reduced in ING4 overexpression cells, while increased in the ING4 knockdown cells, when compared with relevant controls (Figure 5B). Simultaneously, Real time PCR validated that ING4 could regulate p21 mRNA expression in spite of p53 expression, which was consistent with the finding in the protein levels (Figure 5C). Moreover, the reporter gene assay also showed that p21 promoter could be significantly activated by ING4 overexpression whereas attenuated by ING4 knockdown when compared with the controls (Figure 5D).

To further confirm the role of p21 in ING4 mediated Sp1 instability, we used p21 overexpression plasmid in ING4 knocked down p53^+/+^HCT116 cells and western blot was used to test the expressions of ING4, p21, and Sp1. Our data revealed that increased Sp1 expression in the ING4 knocked down cells was distinctly blocked by p21 overexpression (Figure 5E).

## DISCUSSION

The reduced expression of tumor suppressor ING4 has been reported in various malignancies, and down-regulation of ING4 is correlated with increased tumor metastasis, advanced TMN stages and poor patients survival in melanoma [18], gastric cancer [19] and breast cancer [20]. In the present study, we showed that both ING4 mRNA and protein expressions were lower in CRC tissues when compared with paired normal colon tissues. Moreover, low ING4 expression was significantly associated with increased lymph node metastasis, advanced TNM stage and poor overall survival. In addition, univariate and multivariate Cox regression analyses showed that low ING4 expression was an independent unfavourable prognostic factor of CRC, which were consistent with the report of *Qi et al.* that there is a decreased risk of CRC patient death relative to increased ING4 expression levels [13]. These findings indicated that ING4 might play an important role in CRC progression.

Tumor progression is characterized by tumor cells infinite proliferation and metastasis [21]. And solid tumor lesions exceed a few millimeters in diameter, hypoxia and nutrient deprivation triggers an ‘angiogenic switch’ to satisfy requirements for tumor progression [22]. Therefore, angiogenesis has been described as a key rate-limiting step in tumor proliferation and metastasis [23, 24]. Inhibition of angiogenesis has been shown to prevent tumor progression and produce improved outcomes in patients with metastatic CRC [25, 26]. Angiogenesis is a complex process, which involves the formation of new vessels from the preexisting blood vessels [27]. In this study, we found that tube formation by HUVECs was inhibited in conditioned medium collected from ING4 overexpressing CRC cells, and this effect was confirmed by ING4 knockdown experiments as silencing ING4 in CRC cells resulted in enhanced tube formation ability. Furthermore, ING4 overexpression in CRC cells inhibited the supportive vasculature *in vivo*. These data are consistent with the finding that ING4 loss is correlated with increased microvessel density in colon cancer [14], and partially explained our findings in CRC patient cohort that low ING4 expression in cancer correlated with increased metastasis and poorer outcome in CRC patients.

Transcriptional factor Sp1 plays an important role in tumor-associated angiogenesis due to the regulation of downstream pro-angiogenic genes, such as MMPs and COX-2 [7]. It is well-known that activated MMP-2 contributes to the angiogenic process by remodeling the basement membrane to allow sprouting and liberating matrix-bound angiogenic factors [28]. In addition, COX-2 overexpression in colon carcinoma cells increases their angiogenicity, as shown by an increased ability to stimulate endothelial cell migration and tube formation by producing prostaglandins such as prostaglandin E2 and inducing pro-angiogenic factors such as basic fibroblast growth factor [29–31]. In our study, we found that ING4 significantly reduced the mRNA and protein expressions of MMP-2 and COX-2, which was consistent with the findings in melanoma [18], osteosarcoma [32] and brain cancer [33], and the expression of nuclear Sp1 and subsequent DNA binding activity. Moreover, the role of Sp1 in ING4 regulated CRC angiogenesis was further confirmed by the knockdown of both ING4 and Sp1 together. We finally verified this signal pathway as ING4-knockdown-alone increased MMP-2 and COX-2 expressions, number of tube formation by HUVECs, and neovessels in matrigel plug were abrogated by knockdown of Sp1. Collectively, these results indicated that Sp1 activation is critical for ING4 regulated CRC angiogenesis.

Sp1 has been widely considered to be modified by phosphorylation, sumoylation, acetylation, glycosylation [8]. These post- translational modifications affect not only Sp1 DNA-binding activity, but also its stability through modulation of ubiquitin-proteasomal-dependent degradation [7]. In this study, we found that ING4 did not affect Sp1 mRNA expression, but promoted destabilization of Sp1 and its ubiquitin degradation, suggesting that ING4 can suppress Sp1 expression and transcriptional activity by regulating these post-modifications.

A number of serine/threonine residues of Sp1 protein have indicated that phosphorylation is probably important for its function regulation [7]. Phosphorylated Sp1 level is modulated throughout the cell cycle by different cell cycle proteins [15]. Studies have reported that cyclin/CDK2 complexes and their inhibitors p21 and p27 can mediate phosphorylation of Sp1 [15, 34]. In this study, we found that cell cycle protein ING4 regulated p21 expression to dramatically inhibit the activity of cyclin/CDK2 complexes [17]. These data demonstrated that ING4 might potentially regulate p21-reduced activity of cyclin/CDK2 complexes to cause dephosphorylation of Sp1 and trigger its degradation.

Studies have displayed that INGs can regulated p21 expression through both p53 dependent and independent pathways [10, 16]. In order to determine whether the effect of p21 on ING4-mediated Sp1 dependent on p53 status, the p53-wild and p53-deficient CRC cells were used. It's interesting that regardless of p53 status, ING4 positively regulated p21 expression mRNA and protein levels through the induction of p21 promoter activation, which was accordance with the previous report [35]. Simultaneously, we also found that ING4 reduced the expressions of Sp1 and cyclin-CDK2 complex phosphorylation activity in spite of p53 expression. Moreover, the role of p21 in ING4 regulated Sp1 was further confirmed by ING4 knockdown and p21 overexpression together. We found that ING4-knockdown-enhanced Sp1 expression was abrogated by overexpression of p21. These results indicated that ING4 could regulate p21 expression to suppress Sp1 in spite of p53 expression.

Taken together, reduced ING4 expression in CRC was significantly correlated with metastasis and poorer overall survival of CRC patients, which can be explain at least, in part by enhanced angiogenesis. ING4 suppressed CRC angiogenesis by inhibition of Sp1 expression and transcriptional activity through destabilization and ubiquitin degradation and down-regulation of Sp1 downstream pro-angiogenic factors MMP-2 and COX-2. Moreover, ING4 might induce p21 expression to inhibit phosphorylation activity of cyclin/CDK2 complexes to trigger Sp1 degradation despite of p53 status. Based on these, we drew up a novel potential molecular signal pathway (Figure 5F). Combined with the previous reports about ING4, we propose that ING4 may serve as a promising prognostic marker for CRC, and restoration of ING4 may be a novel strategy for the treatment of metastatic CRC.

## MATERIALS AND METHODS

### Patients and specimens

This study was granted by Institutional Review Board of Affiliated Hospital of Xuzhou Medical University prior to this study. All specimens were obtained before the patients provided their written informed consent. Total ten pairs of fresh CRC and the matched normal tissues were obtained from the surgery department of Affiliated Hospital of Xuzhou Medical University. Simultaneously, a retrospective CRC cohort enrolled 573 cases underwent CRC radical surgery at Affiliated Hospital of Xuzhou Medical University from 2010.4 to 2015.3. The tissues including the CRC tissues and paired non-tumor colon tissues were collected from Pathology Department of Affiliated Hospital of Xuzhou Medical University and constructed into TMAs. The patients' informations including sex, age, marriage, birth place, surgery date, tumor location, tumor differentiation, tumor diameter, depth of invasion, lymph node metastasis, and TNM stage were obtained from the Medical Record of the Affiliated Hospital of Xuzhou Medical University. The overall survival was the primary endpoint of this analysis, survival time was calculated from the date of surgery to the date of death or to the last follow-up. Date of death was obtained from patient records or patients' families through telephone calls and verified by local civil affairs department.

### Construction of tissues microarrays (TMAs) and immunohistochemistry (IHC)

The CRC TMAs were created by contract service at the National Engineering Center for Biochip, Shanghai, China. The paraffin primary tumor and corresponding non-tumor tissue blocks were punched to 1.5mm diameter cores. A standard protocol for the immunostaining of the TMAs was used as described previously [36]. The polyclonal rabbit anti-ING4 antibody (1:2000 dilution, Sigma, USA) was used for primary antibody incubation at 4°C overnight. The slide without primary antibody incubation was used as negative control.

### Assessment of IHC

Due to lost samples during antigen retrieval, no tumor cells present in the core, finally ING4 staining scores were evaluated in 452 non-tumor tissues and 509 CRC tissues. The staining scores of ING4 in the tissues were evaluated independently by two pathologists blinded to the clinical data, by applying a semiquantitative immunoreactivity score (IRS) which was the product of intensity of staining and percentage of immunopositive cells [37]. The intensity of ING4 immunostaining was scored as 0–3 (0, negative; 1, weak; 2, moderate; 3, strong) (Supplementary Figure S2); the percentage of immunoreactive cells was graded as 1 (0–25%), 2 (26–50%), 3 (51–75 %) and 4 (76–100%). The concordance for IRS staining score of ING4 between the two pathologists was 430 in 452 non-tumor colon tissues (95%) and 479 in 509 tumors (94%), and the few discrepancies were resolved by scanning TMA pictures (Aperio Scanscope FL).

The receiver-operator characteristic (ROC) analysis was performed to obtain the optimum cutoff value of ING4 IRS, and the areas under the curves at different cutoff values of the ING4 IRS for 1, 3 and 5 years of overall survival time were calculated. The optimum value of cutoff point of the ING4 IRS was shown to be 3 since it had the best predictive value for survival (Supplementary Figure S3). Under these conditions, samples with IRS 0–3 and IRS 4–12 were classified as low or high expression of ING4.

### Western blotting and antibodies

Cytoplasmic and Nnuclear extracts were obtained using the NE-PER Nuclear and Cytoplasmic Extraction Reagents (Pierce Biotechnology, USA) following with the manufacturer's instructions. Total cell lysates were prepared with a detergent lysis buffer. Western blots were carried out as previously reported [36, 37]. The rabbit anti-ING4 (1:2000, Abcam, USA), anti-MMP-2 (1:1000, Cell Signaling Technology, USA), anti-MMP-9 (1:1000, Cell Signaling Technology, USA), anti-VEGF (1:1000, Abcam, USA), anti-COX-2 (1:5000, Abcam, USA), anti-Sp1 (1:5000, Abcam, USA), anti-histone H3 (1:2000, Abcam, USA), anti-ubiquitin (1:1000, Abcam, USA), anti-cyclinA (1:1000, Cell Signaling Technology, USA), anti-cyclinE (1:1000, Cell Signaling Technology, USA), anti-CDK2 (1:2000, Abcam, USA), anti-p21 (1:1000, Cell Signaling Technology, USA), anti-p27 (1:1000, Cell Signaling Technology, USA), anti-histone H1 (1:2000, Abcam, USA) and anti-p53 (1:1000, Santa Cruz Biotechnology, USA) were used for primary antibody incubation at 4°C overnight. The mouse anti-α-tubulin (1:1000, beyotime institute of biotechnology, China) was used for the protein loading control. Each blot was repeated at least three times. The intensity of the protein bands were analyzed by densitometry after normalization to the corresponding protein controls.

### Real time PCR

Total RNA was extracted from the cells and tissues using the Trizol reagent (Invitrogen). Approximately 1 μg of RNA was used for the reverse transcription reaction using the PrimeScript™ RT reagent Kit with gDNA Eraser (Vazyme). The cDNA was amplified with the following primers: 5′-CACAGACCTGGCCCGTTTT-3′ (forward) and 5′- AGTCCGGCCTTTCTTTTTGC-3′ (reverse) for ING4; 5′-CTTCCAAGTCTGGAGCGATGT-3′ (forward) and 5′-TACCGTCAAAGGGGTATCCAT-3′ (reverse) for MMP-2; 5′-GTACTCGACCTGTACCAGCG-3′ (forward) and 5′-AGAAGCCCCACTTCTTGTCG-3′ (reverse) for MMP-9; 5′-TCTCGACATCGAGGACCCAT-3′ (forward) and 5′-TGGACCAGTCGAAACCCTTG-3′ (reverse) for TIMP-2; 5′-CTGTCTAATGCCCTGGAGCC-3′ (forward) and 5′-ACGCGAGTCTGTGTTTTTGC-3′ (reverse) for VEGF; 5′-CTGGCGCTCAGCCATACAG′ (forward) and 5′-CGCACTTATACTGGTCAAATCCC′ (reverse) for COX-2*;* 5′-GGTGCCTTTTCACAGGCTC'-3′ (forward) and 5′-GCTGTTCTCATTGGGTGACTC-3′ (reverse) for Sp1; 5′-GCCGGTGCTGAGTATGTC-3′ (forward) and 5′-CTTCTGGGTGGCAGTGAT-3′ (reverse) for GAPDH.

Real time PCR was carried out in triplicate with SYBR Green PCR Master Mix using a 7900HT qPCR system thermal cycler (Applied Biosystems) as described previously [37]. GAPDH mRNA was used as an internal control for each sample, and the Ct value for each sample was normalized to GAPDH mRNA.

### Animals and cell lines

This experiment *in vivo* was approved by the Animal Care Committee of Xuzhou Medical University. Female BALB/c nude mice, 6–8 weeks old, were purchased from NLARSH China (Shanghai, China), and maintained under specific pathogen-free conditions. Human CRC cell lines p53^+/+^HCT116, p53^−/−^HCT116 and HCT15 were obtained from the Shanghai Institute of Biochemistry and Cell Biology, Chinese Academy of Sciences (Shanghai, China). These two cell lines were cultured 1640 medium supplemented with 100 U/ml penicillin, 100 μg/ml streptomycin and 10% fetal bovine serum and incubated in a 37°C humidified incubator with 5% CO_2_.

### Plasmids, siRNA and transient transfections

The HA-Vector (HA-Vec), HA-ING4 (ING4 overexpression plasmid), pcDNA-EmGFP-miR-Vector (mi-Vec) and pcDNA-EmGFP-miR-ING4 (mi-ING4, ING4 knockdown plasimd) plasmids were kindly provided by Dr. Li Gang (University of British Columbia) [38]. The p21 cDNA construct was derived from Flag-p21 plasmid (Addgene), then inserted into pEGFP-C1 construct (Clontech) to produce pEGFP-C1-p21 [39]. The pGL2-p21 promoter-Luc luciferase-reporter plasmid was purchased from Addgene and the pRL-SV40 and GFP-ub plasmids were used as reported previously [37, 40]. All the plasmids were confirmed before using by DNA sequencing. The specific siRNA for Sp1 (si-Sp1) and non-specific control (si-Vec) were purchased from Santa Cruz Biotechnology (Cat.No.sc-29487). For plasmid and siRNA transfection, CRC cells were grown to approximately 50% confluency and then transiently transfected with plasmids and siRNA using lipofectamine 2000 transfection reagent (Invitrogen), respectively, according to the manufacturer's instructions. Twelve hours after transfection, the medium containing transfection reagents was removed, and the cells were incubated in fresh medium.

### Tube formation assay *in vitro* and *in vivo* angiogenesis assay

1 **×** 10^6^ ING4 over-expressed or knocked down CRC cells and respective control cells were cultured in 60-mm plates with 2 ml fresh serum-free medium for 24 hours, then 24 hours' conditioned medium was collected. For tube formation assay, the 96-well plate was coated with 50 μl matrigel ™ (BD Biosciences) and kept at 37°C for 2 hours. 1 × 10^4^ HUVECs were suspended in 100μl conditioned medium and seeded into the pre-coated 96-well plate and cultured for 12 hours, photos were taken under a microscope, and the complete tubular structures were counted. For *in vivo* angiogenesis assay, every 3 BALB/c nude mice were randomly divided into a group. p53^+/+^HCT116 and HCT15 (5 × 10^6^) cells that transiently transfected with HA-Vec (control) or HA-ING4 (ING4 overexpression) plasmids for 24 hours and p53^+/+^HCT116 cells that were transfected with mi-Vec (control) or mi-ING4 (ING4 knockdown) together with or without Sp1 siRNA (si-Sp1) for 24 hours were supported by 200μl and subcutaneously implanted into the flanks of 6–8 week old male nude mice, respectively. Ten days later, the mice were killed and the implanted matrigel plugs were excised and photographed.

### Electrophoretic mobility shift assays (EMSA)

EMSA was performed with a Biotin Gel Shift Kit (Pierce Biotechnology, USA). Briefly, 1 μg nuclear extracts from the ING4 overexpressed (HA-ING4), knocked down (mi-ING4), and control (HA-Vec and mi-Vec) p53^+/+^HCT116 were added into a mixture containing 40 fmol of biotin 5′-endlabeled, double-stranded probes (5′-ATTCGATCGGGGCGGGGCGAGC-3′) (Invitrogen) bearing Sp1 consensus binding sequence in 20 μl of binding buffer, respectively. For super shift EMSA, 1 μg Sp1 antibody or control IgG was added to the extract from HA-Vec cells, and incubated for 20 min on ice before addition of the reaction mixture. For the competition reaction, 1μl nuclear extract from HA-Vec cells was added to mixtures contained a 100-fold the same sequence but non-labeled double-stranded oligonucleotide. The detection procedure was carried out as described previously [37].

### Coimmunoprecipitation (IP)

IP assay was performed following the standard procedures described previously [40]. Precleared lysates were incubated with Sp1 antibody for 2 hours, and then protein A/G agarose beads (Santa Cruz Biotechnology) were added into these lysates to incubate at 4°C overnight. The beads were collected by centrifugation, washed 5 times with wash buffer and resuspended in 1 **×** sodium dodecyl sulfate loading buffer. The immunoprecipitates were eluted from the beads by incubation at 95°C for 5 min. The eluted proteins were separated by sodium dodecyl sulfate polyacrylamide gel electrophoresis, and western blot was subsequently performed with antiubiquitin antibody.

### Luciferase reporter gene assay

Cells were seeded onto 24-well plates (5 × 10^4^ cells per well), and co-transfected with 0.5 μg pGL2-p21 promoter-Luc luciferase-reporter plasmid, 10 ng pRL-SV40, ING4 overexpression (HA-ING4) or ING4 knockdown (mi-ING4) or control plasmids (HA-Vec and mi-Vec) for 24 hours, then cell lysates were prepared according to Promega's instruction manual. Luciferase activity was measured with a dual-Luciferase Reporter Assay System (Promega, USA) and the activity was normalized against the Renilla luciferase gene.

### Statistical analysis

The significance of ING4 staining in cancers and their corresponding non-tumor colon tissues was assessed by the paired Wilcoxon test. The association between ING4 expression and clinicopathological parameters was evaluated by Fisher's exact test. Probability of differences in overall survival as a function of time was ascertained by Kaplan–Meier method and log-rank test. Univariate and multivariate Cox proportional hazards regression analyses were performed to estimate the crude hazard ratios (HRs), adjusted HRs and 95% confidence intervals (CIs) of HRs. All the statistical analyses were performed by STATA statistical software (version 12.1; StataCorp, College Station, TX). *P* value < 0.05 was deemed statistically significant, and all tests were two sided.

## SUPPLEMENTARY MATERIALS


