# Supplementary material for: ING4 suppresses tumor angiogenesis and functions as a prognostic marker in human colorectal cancer
Source: Oncotarget. 2016 Oct 27;7(48):79017–31. doi: 10.18632/oncotarget.12984 (PMC5346695; doi:10.18632/oncotarget.12984)
Supplement: Supplementary file 1 [file oncotarget-07-79017-s001.pdf]

ING4 suppresses tumor angiogenesis and functions as a prognostic marker in human colorectal cancer

Supplementary Materials

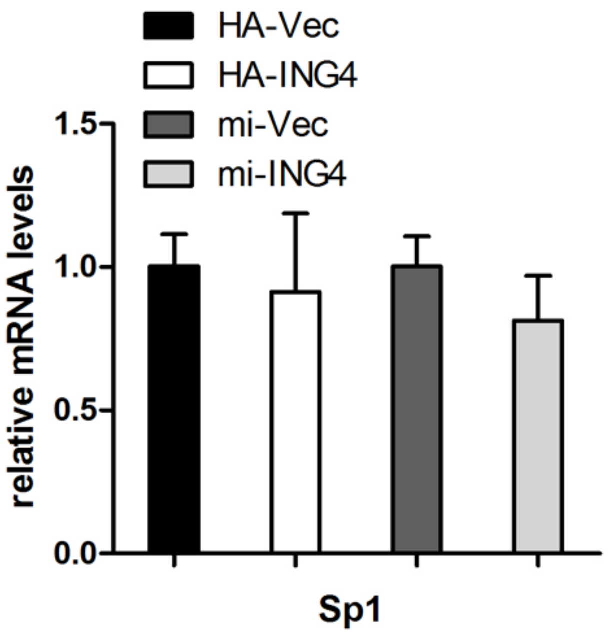

Supplementary Figure S1: The real time PCR confirmed that ING4 had no effect of Sp1 mRNA expression in $p53^{+/+}$HCT116 cells.

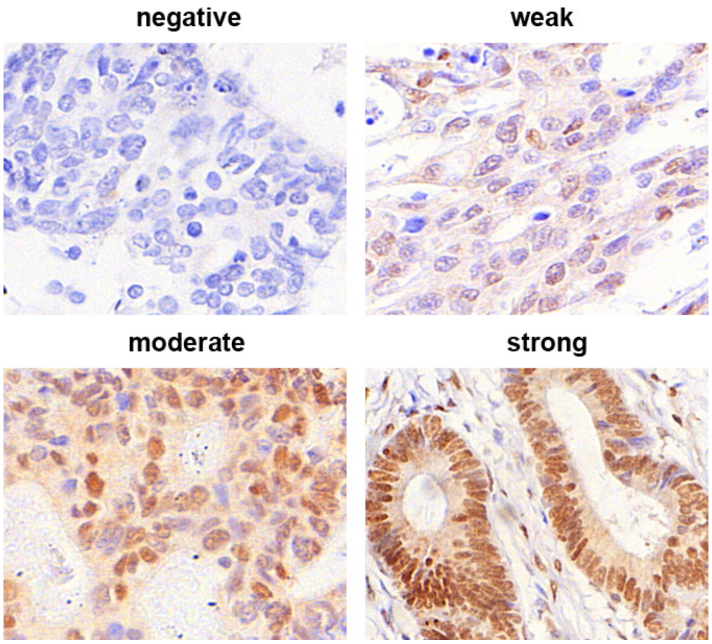

Supplementary Figure S2: Representative images of ING4 immunohistochemical staining in human CRC tissues. Note: original magnification, × 400.

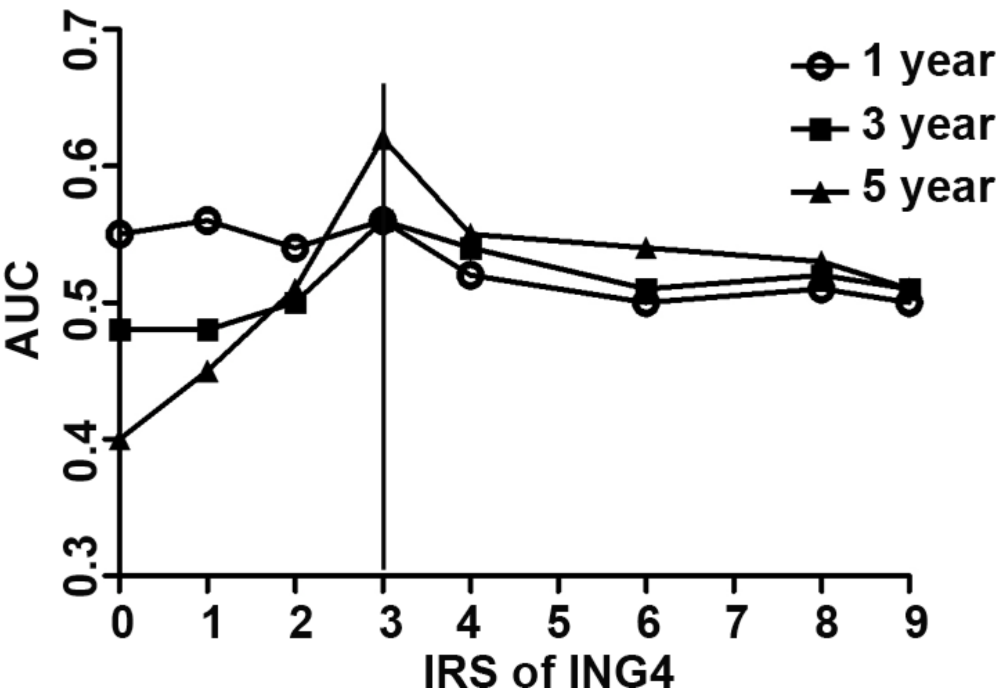

Supplementary Figure S3: ROC is obtained to determine the optimal cutoff value of ING4 expression. ROC obtains the area under the curves (AUCs) at different cutoff values of ING4 IRS for 1, 3 and 5 years of overall survival time
